# Effect of Roux-en-Y Gastric Bypass on the NLRP3 Inflammasome in Adipose Tissue from Obese Rats

**DOI:** 10.1371/journal.pone.0139764

**Published:** 2015-10-05

**Authors:** Andreea Oana Mocanu, Anny Mulya, Hazel Huang, Olivia Dan, Hideharu Shimizu, Esam Batayyah, Stacy A. Brethauer, Anca Dinischiotu, John P. Kirwan

**Affiliations:** 1 Biochemistry Department, Faculty of Biology, University of Bucharest, Bucharest, Romania; 2 Department of Pathobiology, Cleveland Clinic, Cleveland, Ohio, United States of America; 3 Department of Bariatric Metabolic Institute, Cleveland Clinic, Cleveland, Ohio, United States of America; 4 Department of Gastroenterology & Hepatology, Cleveland Clinic, Cleveland, Ohio, United States of America; 5 Metabolic Translational Research Center, Cleveland Clinic, Cleveland, Ohio, United States of America; University College Dublin, IRELAND

## Abstract

**Objective:**

Obesity is associated with low-grade chronic inflammation. We hypothesized that Roux-en-Y gastric bypass (RYGB) surgery would reduce activation of the NLRP3 inflammasome in metabolically active adipose tissue (AT) of obese rats, and this change would be related to decreases in body weight and improved glycemic control.

**Methods:**

Omental, mesenteric and subcutaneous fat depots were collected from Sprague-Dawley rats: Sham control and RYGB; 90-days after surgery. NLRP3, caspase–1, apoptosis-associated speck-like protein (ASC), IL–1β, IL–18, IL–6 and MCP–1 gene and protein expression were quantified. Glucose metabolism was assessed by oral glucose tolerance test (OGTT).

**Results:**

Compared to Sham surgery controls, RYGB surgery decreased IL–6, MCP–1, NLRP3, IL–18, caspase–1 and ASC in omental fat, and decreased IL–6, MCP1, IL–1β, IL–18, caspase–1 and ASC gene expression in mesenteric fat. We observed differential gene expression between visceral and subcutaneous fat for IL–6 and IL–1β, both being downregulated by RYGB in visceral, and upregulated in subcutaneous depots. These changes in gene expression were accompanied by a decrease in NLRP3, ASC, IL–18, caspase–1 and IL–1β protein expression in omental tissue. We found a positive correlation between caspase–1, ASC, MCP–1, IL–18 and IL–6 gene expression following surgery and glucose AUC response in omental fat, while the change in glucose AUC response correlated with caspase–1 gene expression in subcutaneous fat.

**Conclusion:**

This study demonstrates that bariatric surgery reverses inflammation in visceral adipose tissue by suppressing NLRP3 inflammasome activation. These are the first data to implicate the NLRP3 inflammasome in diabetes remission after RYGB surgery.

## Introduction

Obesity is characterized by massive expansion of adipose tissue (AT) and is closely associated with a chronic, low-grade inflammatory state and insulin resistance, which conspire to increase the risk of type 2 diabetes and related morbidity and mortality. Obesity-associated inflammation occurs as a result of immune cell infiltration into the adipose tissue and increased production of pro-inflammatory cytokines [1] such as IL–1β, IL–6 and TNF-α, leading to the pathogenesis of insulin resistance and eventually to the development of type 2 diabetes. The mechanisms by which obesity leads to the pro-inflammatory state are not well understood.

Nod-like receptor family, pyrin domain containing 3 (NLRP3), a pattern recognition receptor, that can form a multiprotein inflammasome complex, may play an important role in initiating the inflammatory response. Upon its activation, NLRP3 induces the recruitment and the autocatalytic activation of the cystein protease caspase–1 that leads to the formation of an inflammasome complex mediated by apoptosis-associated speck-like protein (ASC) [2–7]. The formation of NLRP3 inflammasome and the activation of caspase–1 facilitates the processing of the cytosolic precursor of IL–1β and IL–18 allowing secretion of these biologically active cytokines [8, 9]. The role of the NLRP3 inflammasome in the pathogenesis of obesity-induced insulin resistance is derived from observations that NLRP3 deficient mice fed a high fat diet are more insulin sensitive than HF-diet fed wild-type mice [10]. Further observations by Stienstra et al. [11] demonstrated that NLRP3 inflammasome-mediated caspase–1 activation is an important regulator for adipocyte differentiation and contributes to impaired insulin sensitivity associated with obesity. Further, pharmacological inhibitors or siRNA targeted for caspase–1 or NLRP3, improved insulin sensitivity and adipocyte differentiation. White adipose tissue (WAT) of obese mice show an increase in the activity of caspase–1, IL–1β and IL–18, while caspase–1 deficient mice have smaller adipocytes, lower percentage of total fat mass, increased mitochondrial energy dissipation in WAT and profoundly improved insulin sensitivity [11]. Calorie restriction in mice and patients with type 2 diabetes who lose weight show reduced IL–1β and NLRP3 mRNA in adipose tissue, and this is associated with a decrease in their pro-inflammatory profile and insulin sensitivity [12], [13]. Vandanmagsar et al. further identified the roles of NLRP3 inflammasome in sensing obesity associated danger signals, DAMPS, that contribute to obesity-induced inflammation and insulin resistance [12].

Current therapies for obesity-induced type 2 diabetes are limited. Lifestyle interventions that include diet and exercise as well as pharmacological therapy, work to varying extents, but the results tend to be short-lived. Bariatric surgery has profound metabolic effects and restores glycemic control in patients with morbid obesity and/or type 2 diabetes [14], [15], [16], [17], [18], [19], [20]. It has been previously demonstrated that bariatric surgery improves long-term weight loss and is accompanied by a reduction in WAT pro-inflammatory state [11], is associated with a reduction of subcutaneous adipose tissue macrophage infiltration and down-regulation of inflammatory cytokines, such as TNF-α and IL–6 [12], [13]. However, little is known about the effects and mechanisms of bariatric surgery on the inflammatory state of visceral fat, and its association with improvement in weight loss and whole body insulin sensitivity post intervention. We hypothesized that RYGB surgery reduces the inflammatory profile (“low-grade” inflammation) determined by the down-regulation of the NLRP3 inflammasome in adipose tissue from obese rats and that this change is positively associated with reduced body weight and improved glycemic control.

## Materials and Methods

### Animal Care

The protocol was approved and performed in compliance with the Cleveland Clinic Institutional Animal Care and Use Committee (IACUC). Sixteen 12-week old Sprague-Dawley (SD) rats (Charles River Laboratories, Wilmington, MA) were included in this investigation. Rats were housed in individual cages, kept at a constant temperature and ambient humidity in a 12-hour light/dark cycle. Animals were provided an *ad libitum* high-fat (D12492, 60% fat, Research Diets, New Brunswick, NJ) diet for 12 weeks to establish diet-induced obesity, hyperglycemia and insulin resistance. At age 24–25 weeks, the animals were randomized into a control sham-operated (N = 8), or RYGB (N–8) surgery group.

### Surgical Intervention

All animals were fasted overnight (approximatively 16 hours). Ceftriaxone 75 mg/kg was administered intramuscularly for prophylaxis, an isoflurane gas chamber was used for anesthesia during the procedure. We used the gastric bypass model and bowel limb lengths as previously described [21], and as proposed by Meguid et al. [22] to achieve durable weight loss. For the sham operations a midline incision was made, and the stomach and distal esophagus were exposed; the small bowel was laid out for the same duration required for gastric bypass procedures. The abdomen was closed in layers with sutures. The rats were maintained on an *ad libitum* liquid diet with Boost (Nestle, Buffalo Grove, IL) for up to 7-days after surgery. Thereafter, they were fed a HFD *ad libitum*. All rats were euthanized on postoperative day 90. Adipose tissues from three different depots, omentum (OM), mesenteric (MS) and subcutaneous (SQ), were collected, snap frozen in liquid nitrogen, and stored at -80°C.

### Glucose Tolerance Test

A fasting oral glucose tolerance test (OGTT) was performed in rats preoperatively and repeated on day 32 post surgery, by measuring the blood glucose response via tail vein puncture at baseline, 10, 20, 30, 60, 90, 120, 150, and 180 minutes after oral gavage with a 3.0 g/kg glucose solution (70% dextrose solution). Glucose area under the curve (AUCs) was determined by the trapezoidal method.

### Total RNA Extraction

Total RNA was extracted from the adipose tissues using the RNeasy Lipid Tissue Mini Kit (Qiagen, Valencia, CA) according to the manufacturer’s instruction. Briefly, 50–100 mg of AT tissue was homogenized in β-mercaptoethanol and QIAzol lysis reagent. The homogenate was separated into aqueous and organic phase by adding chloroform followed by centrifugation. Ethanol was added to the collected upper aqueous phase to provide optimized RNA binding condition to the RNeasy mini spin column. Genomic DNA was removed by adding DNase following the protocol of RNase-free DNase kit (Qiagen) by adding the DNase to the column, followed by 30 minute incubation at room temperature. Total RNA bound to the column was washed and finally eluted in 50 μl of nuclease free water by centrifugation. RNA concentration and purity was determined from absorbance at 230, 260 and 280 nm using a NanoDrop ND–1000 Spectrophotometer (Thermo Scientific, Wilmington, DE). Isolated RNA was aliquoted and stored at -80°C until further analysis.

### cDNA synthesis

One microgram of cDNA was prepared from total RNA by reverse transcription reaction following the iScript cDNA synthesis kit (Biorad, Hercules, CA) using a PX2 Thermal Cycler (Thermo Scientific). The reaction was set up at a volume of 20 μl and synthesis was performed in the following order, 5 minutes at 25°C, 30 minutes at 42°C and 5 minutes at 85°C. Complementary DNA was stored at -80°C until semiquantitative real time PCR (qRT-PCR) analysis was performed.

### qRT-PCR Primer Pairs

Primer pairs for specific target genes were obtained from PrimerBank database (pga.mgh.harvard.edu/primerbank/) for mouse and checked for the specificity to the genes of interest by conducting a Blast analysis against the rat mRNA sequence (see Table 1).

**Table 1:** Gene specific primers for qRT-PCR analysis.

Gene		Primer Sequence	Amplicon Size (nt)	GeneBank Accession #
MCP–1	Forward	TAGCATCCACGTGCTGTCTC	94	NM_031530.1
	Reverse	CAGCCGACTCATTGGGATCA		
NLRP3	Forward	TTCCCAGACCCTCATGTTGC	306	NM_001191642.1
	Reverse	CAGGGCATTGTCACTGAGGT		
CASP1	Forward	GCCGTGGAGAGAAACAAGGA	319	NM_012762.2
	Reverse	ACCCTTTCAGTGGTTGGCAT		
IL18	Forward	GACCGAACAGCCAACGAATC	84	NM_019165.1
	Reverse	TAGGGTCACAGCCAGTCCTC		
IL1β	Forward	GAGTCTGCACAGTTCCCCAA	88	NM_031512.2
	Reverse	TGTCCCGACCATTGCTGTTT		
IL6	Forward	GCAAGAGACTTCCAGCCAGT	143	NM_012589.2
	Reverse	CCTCCGACTTGTGAAGTGGT		
ASC	Forward	GGACAGTACCAGGCAGTTCG	140	NM_172322.1
	Reverse	GTCACCAAGTAGGGCTGTGT		
GAPDH	Forward	TCAAGAAGGTGGTGAAGCAG	111	NG_028301.2
	Reverse	AGGTGGAAGAATGGGAGTTG		

Gene specific primers for qRT-PCR analysis. MCP–1—monocyte chemoattractant protein–1; NLRP3—NOD-like receptor family, pyrin domain containing 3; CASP1—caspase 1; IL18—interleukin 18; IL1β–interleukin 1-beta; IL6—interleukin 6; ASC—Apoptosis-associated Speck-like Protein Containing a Caspase Recruitment Domain; GAPDH—glyceraldehyde-3-phosphate dehydrogenase; (pga.mgh.harvard.edu/primerbank/)

### Semi-quantitative RT-PCR Analysis

Determination of relative mRNA expression was performed in duplicate on an MX3000P QPCR system (Agilent Technologies/Stratagene, Santa Clara, CA) using 10 ng of cDNA as the template and the VeriQuest SYBR Green qPCR Master Mix (Affymetrix, Santa Clara, CA). Sample normalization was done using the rat GAPDH gene as an internal standard. The relative changes in mRNA abundance were calculated using the comparative ΔΔCt method [23]. Briefly, the threshold cycle (Ct) for GAPDH was subtracted from the Ct for the gene of interest to adjust for variations in mRNA/cDNA generation efficiency to determine the ΔCt value. Fold induction of target genes of RYGB animal was calculated as an exponential of the negative value by the subtraction of ΔCt of an RYGB animal from the average ΔCt of a Sham animal (2^-ΔΔCt^).

### Protein Extraction and Western Blot Analysis

Total protein was extracted from approximately 100 mg of adipose tissue using a glass homogenizer in 1 mL of cold RIPA lysis buffer (20 mM Tris-HCl (pH 7.5), 150 mM NaCl, 1 mM Na_2_EDTA, 1 mM EGTA, 1% NP–40, 1% sodium deoxycholate, 2.5 mM sodium pyrophosphate, 1 mM β-glycerophosphate, 1 mM Na_3_VO_4_) in the presence of protease inhibitor (Sigma Aldrich, St. Louis, MO), 5 mM phenylmethylsulfonyl fluoride (Sigma Aldrich) and Phos-STOP (Roche Applied Sciences, Indianapolis, IN). Protein homogenates were centrifuged at full speed for 20 minutes at 4°C. Fat cake was removed by aspiration and the infranatant was transferred to a new tube. Samples were then aliquoted and stored at -80°C until further analysis. Protein concentration was measured using a Protein BCA assay kit (Pierce Biotechnologies, Rockford, IL). 50 μg of total protein was prepared with Laemmli sample buffer containing 1% of β-mercaptoethanol as reducing agent and boiled for 5 minutes. The protein samples were separated in 4–20% Novex TrisGlycine SDS PAGE Electrophoresis System (Life Technologies, Camarillo, CA), followed by transferring into polyvinylidene fluoride membrane (Biorad). The membrane was blocked for 1h with 5% bovine serum albumin in phosphate buffered saline with 0.1% Tween 20 (PBS-T) at room temperature. Membranes were then incubated overnight with primary antibody at a dilution 1:1000 in PBS-T to recognize specific target proteins MCP–1 (BioLegend, Seattle, WA;Catalog # 505902), NLRP3 (Adipogen, San Diego, CA; Catalog # AG-20B-0014-C100), Interleukin 18 (R&D Systems, Minneapolis, MN; Catalog # AF521), Interleukin 1β (Lifespan Bioscience, Seattle, WA; Catalog # LS-C104781), Interleukin 6 (Abcam, Cambridge, MA; Catalog # ab6672), ASC (Santa Cruz Biotechnology, Dallas, TX; Catalog # sc-22514-R), with GAPDH (Santa Cruz Biotechnology; Catalog # sc–20357) and/or actin (Santa Cruz Biotechnology; Catalog # sc–1616) as loading controls. Membranes were washed with PBS-T and incubated with secondary horseradish peroxidase-conjugated antibodies, for anti-rabbit, anti-mouse (GE Healthcare, Piscataway, NJ) and anti-goat (Santa Cruz Biotechnology). Immunoreactive proteins were visualized by enhanced chemiluminescence reagent (ECL Prime; GE Healthcare, Piscataway, NJ) and quantified by densitometric analysis using ImageJ software.

### Caspase1 Activity Assay

Caspase 1 activity was assessed using a Caspase–1/ICE Fluorometric Assay kit (Biovision Inc, Milpitas, CA) based on the detection of cleavage substrate YVAD-AFC (AFC: 7-amino-4-trifluoromethyl coumarin). The cleavage product, free AFC emits a yellow-green fluorescence (λmax = 505nm). Briefly, adipose tissue was homogenized in chilled lysis buffer provided and 200 μg of total protein lysate was used in the assay. A standard curve was generated using human recombinant active caspase–1 (BioVision Inc.). Lysate or human recombinant active caspase–1 was incubated with reaction buffer (provided) and 50 μM of YVAD-AFC substrate for 2 hours at 37°C. The fluorescence signal was read using a fluorometer equipped with a 400 nm excitation filter and 505 nm emission filter.

### Statistical Analysis

Repeated measure ANOVA analysis was used to determine differences in body weight and the glucose AUC response during OGTT at pre and post-surgery intervention. Statistical differences in gene, protein expression and caspase–1 activity assay between the sham and RYGB groups were analyzed using U Mann-Whitney test. Correlations between gene expression and body weight or OGTT were determined using Pearson correlation analysis. The Grubbs’ test for outlier detection was used to test the presence of outliers, which were excluded from the calculations if analysis was shown to be significant. Data are expressed as mean±SE, the threshold of significance was set at P<0.05.

## Results

### Body weight and glycemic control

**Fig 1:**
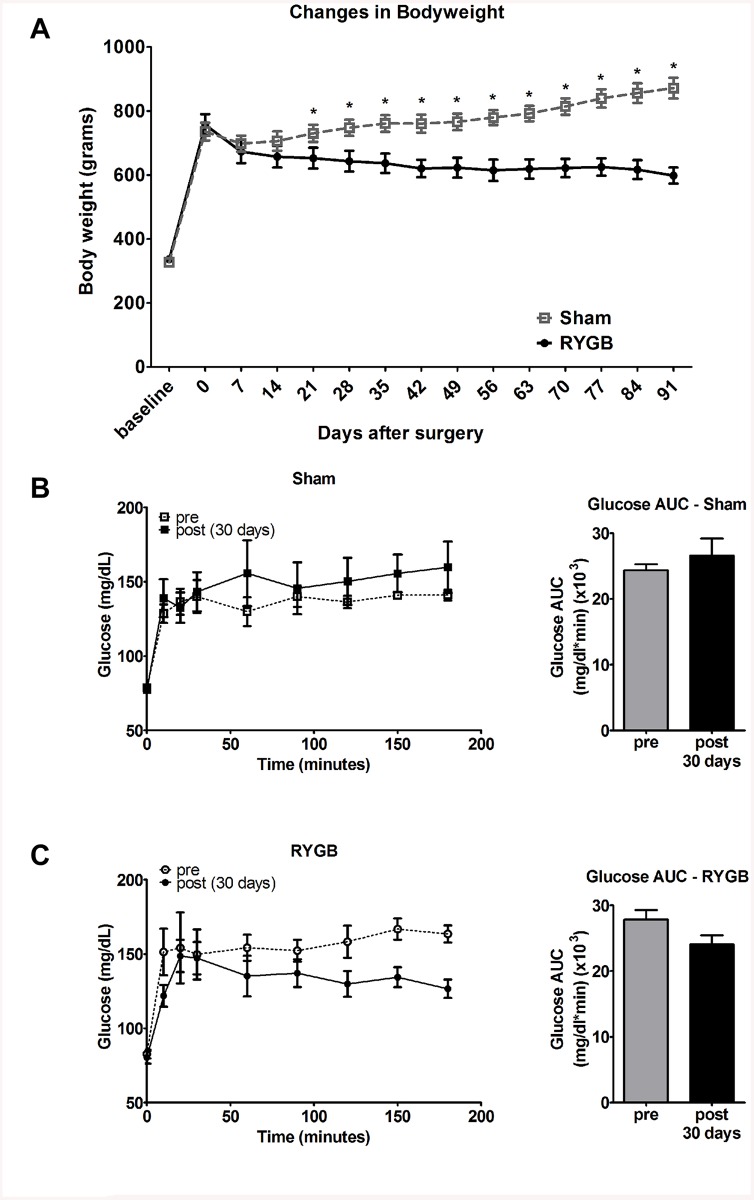
RYGB surgery improves body weight (A) and glycemic control (B) for up to 90 days in SD rats fed a high fat diet. (A) Records of body weight for Sham control vs RYGB animals. (B and C) Plasma Glucose response (left) and glucose AUC (right) during OGTT for Sham (B) and RYGB (C), before (gray) and after (black) surgery. Data are mean ± SE, asterisk sign denotes a significant difference between Sham control vs. RYGB with P<0.05.

Baseline body weight was 709 ± 22 and 762 ± 32 grams for the control, and RYGB groups, respectively. The RYGB group lost 21% of their body weight despite eating the HFD for 90 days. In contrast, the Sham control group showed a 20% weight gain (Fig 1). Repeated measures ANOVA analysis of body weight demonstrated there is an effect of time (*P*<0.0001) and interaction (time x group) between sham and RYGB group (*P*<0.0001). There was no significant difference in daily food intake between Sham (20.6 ± 0.8 grams) and RYGB (23.0 ± 1.5 grams) groups. RYGB surgery improved glycemic control over the Sham control group as demonstrated by glucose AUC response during OGTT (Fig 1). Repeated measure ANOVA also indicated that for the RYGB treated animals there was a statistically significant improvement (decrease) in OGTT AUC (p<0.04), while in the Sham controls did not change (p<0.14).

### Gene expression in omental, mesenteric and subcutaneous adipose tissue

**Fig 2:**
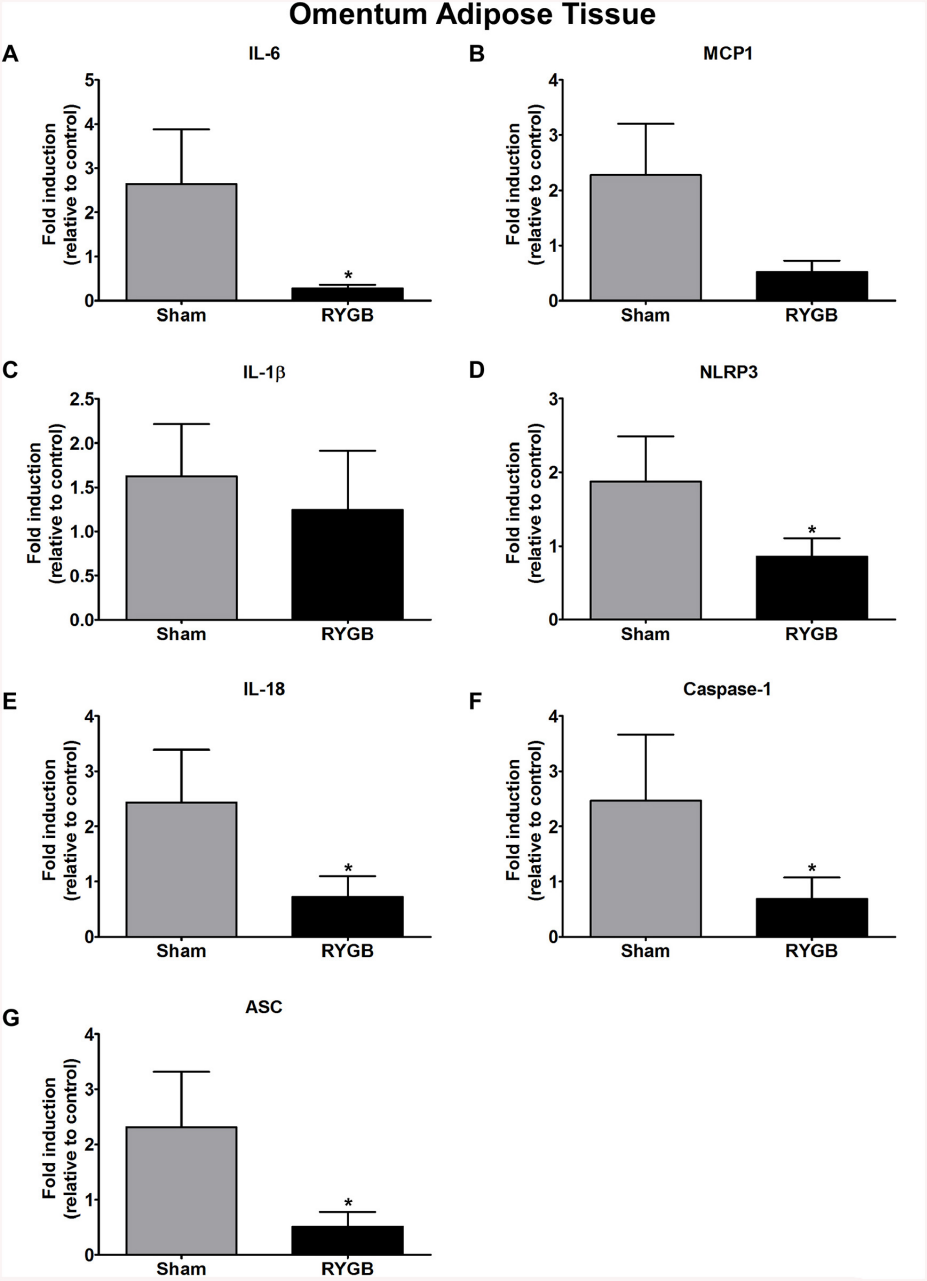
Expression of components of the NLRP3 inflammasome, IL–6 (A), MCP–1 (B), IL–1β (C), NLRP3 (D), IL–18 (E), Caspase–1 (F) and ASC (G) in Sham control (grey bar) and RYGB (black bar) omental adipose tissue. N = 8—SHAM and N = 8—(RYGB), data are mean ± SE, asterisk sign denoted significant different between control vs. RYGB.

**Fig 3:**
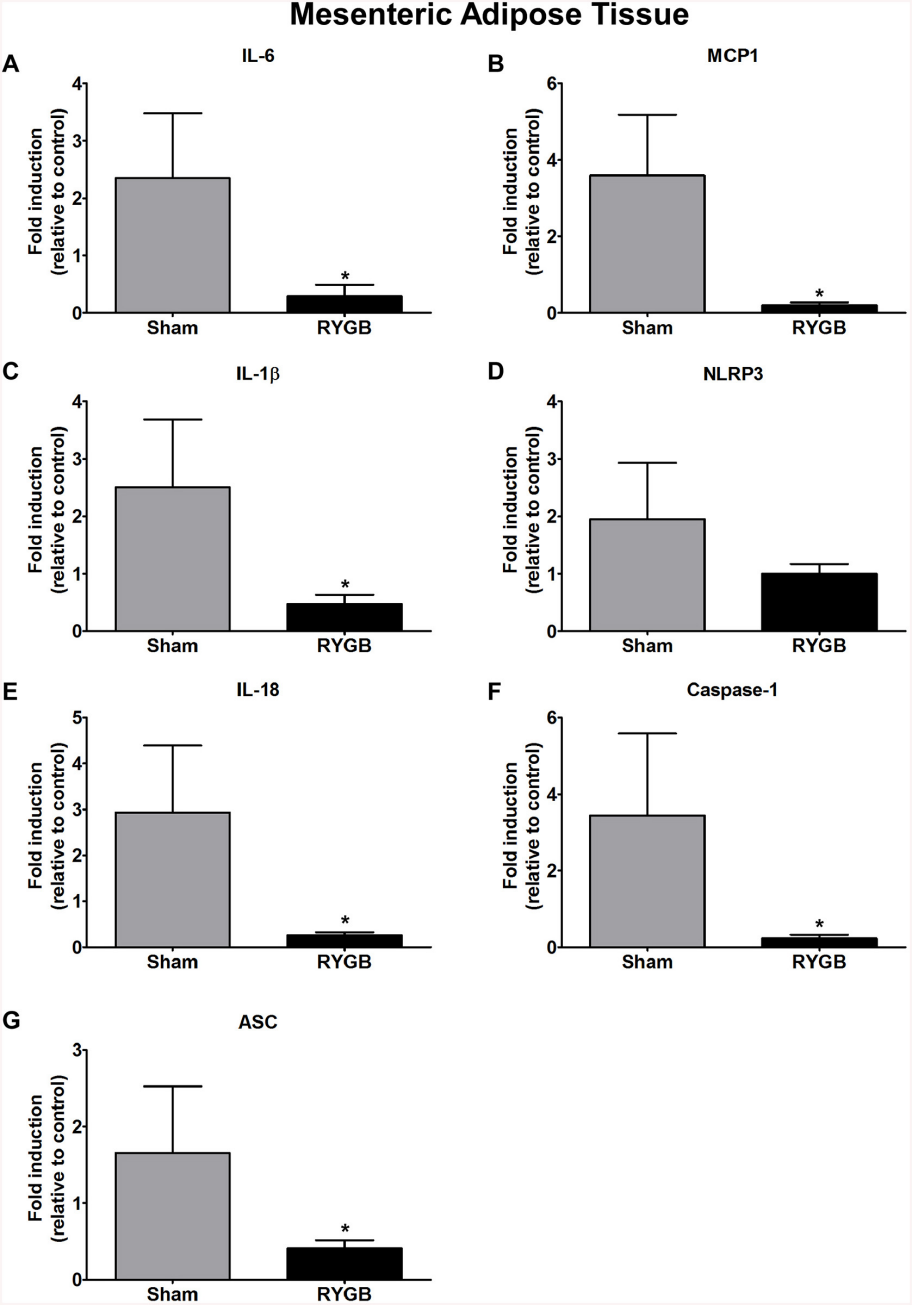
The expression of IL–6 (A), MCP–1 (B), IL–1β (C), NLRP3 (D), IL–18 (E), caspase–1 (F) and ASC (G) genes in mesenteric adipose tissue between sham and RYGB groups. N = 8 (control) and N = 8 (RYGB). Data are mean ± SE. * denotes a significant difference between Sham control vs. RYGB.

**Fig 4:**
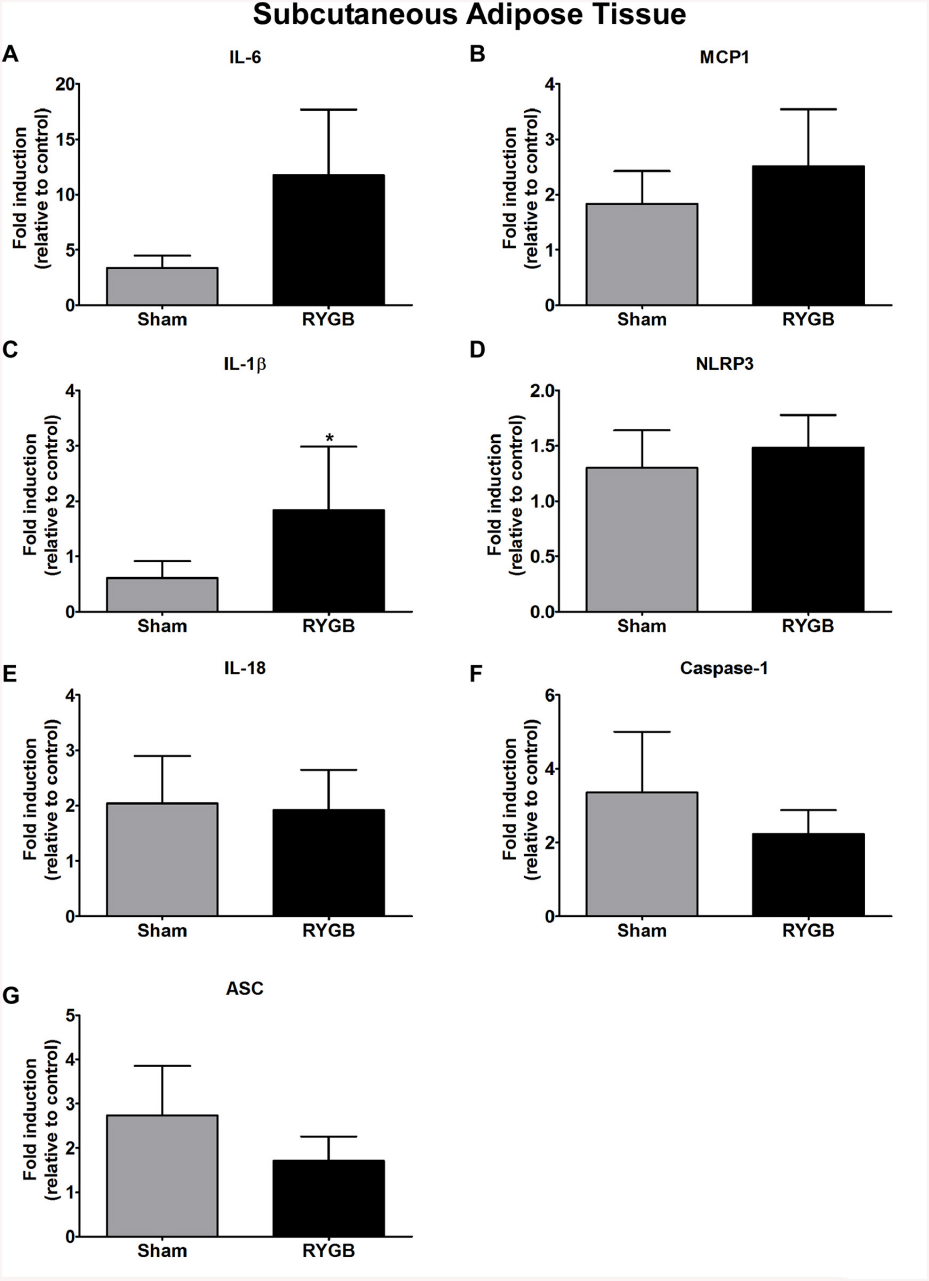
Differential gene response in subcutaneous adipose tissue. Differential gene response of IL–6 (A), MCP–1 (B), IL–1β (C), NLRP3 (D), IL–18 (E), Caspase–1 (F) and ASC (G) in subcutaneous adipose tissue between Sham control and RYGB groups. Data are mean ± SE (N = 8 for both control and RYGB groups), * denotes a significant difference between Sham control vs. RYGB.

The expression of IL–6 (p<0.00008), caspase–1 (p<0.00004), ASC (p<0.00004), NLRP3 (p<0.00009), IL–18 (p<0.0006) and MCP1 (p<0.0001) in omental adipose tissue was significantly decreased in the RYGB group compared to Sham controls; IL–1β levels were similar for both groups (Fig 2). We found a significant decrease in the expression of IL–6 (p< 0.0001), MCP–1 (p< 0.00004), IL–1β (p<0.00013), IL–18 (p< 0.0001), caspase–1 (p<0.00004), and ASC (p<0.00008) mRNA in mesenteric adipose tissue following RYGB treatment (Fig 3). Interestingly, in subcutaneous adipose tissue, only IL–1β (p<0.00004) was significantly increased in the RYGB group. Gene expression of caspase–1, ASC, MCP1, IL–6, NLRP3 and IL–18 remained relatively unchanged in the RYGB compared to the Sham control group (Fig 4).

### NLRP3 inflammasome protein expression in omental adipose tissue following RYGB

**Fig 5:**
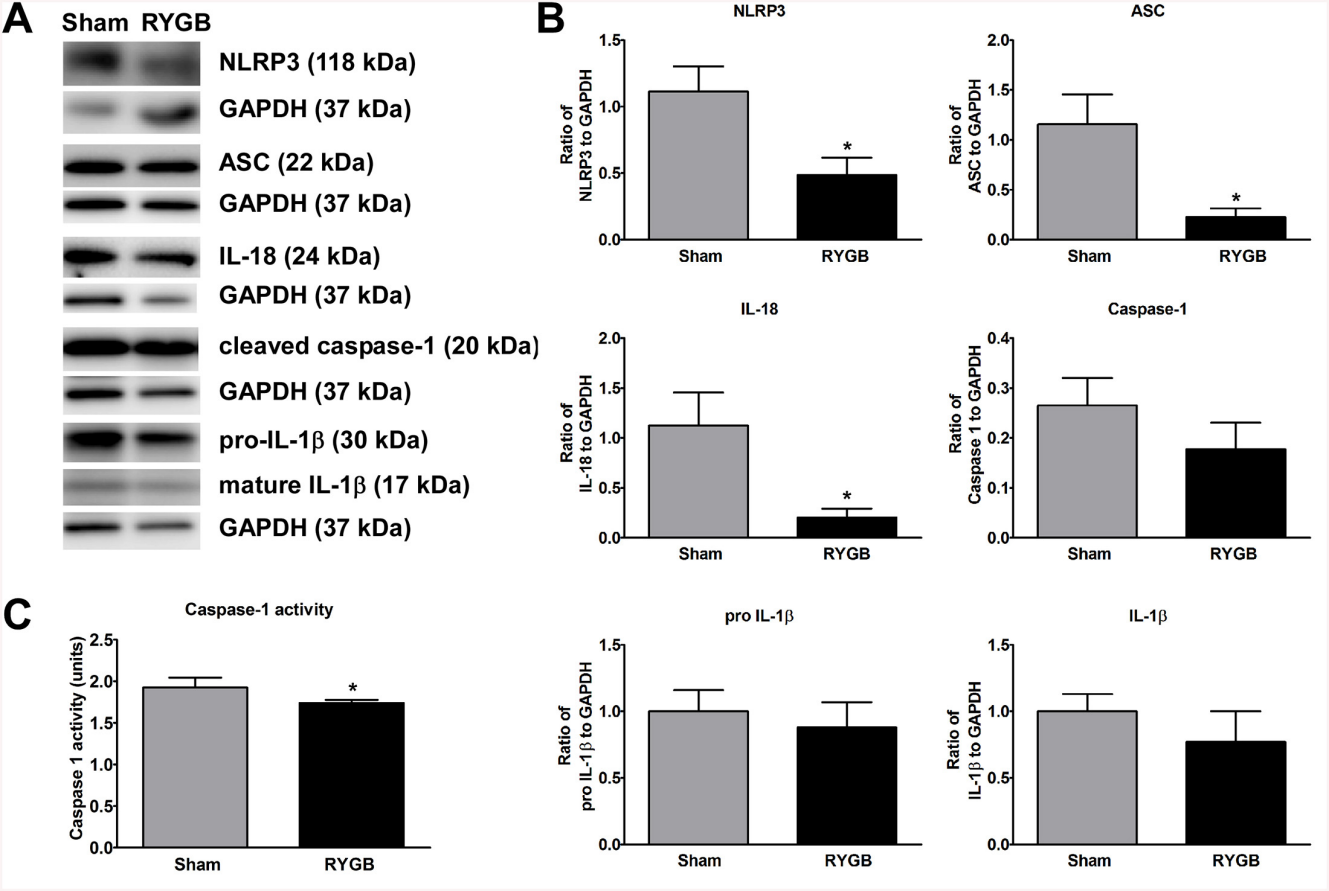
Differential protein expression of NLRP3 inflammasome components in omental adipose tissue. (A) Western blotting representative Western blot images showing NLRP3, ASC, IL–18, cleaved caspase–1, pro-IL–1β and mature IL–1β protein levels (upper) compared with GAPDH loading control (lower). (B) Differential expression of NLRP3, ASC, IL–18, caspase–1, pro-IL–1β and IL-β, control and RYGB in omental adipose tissue. Tissue homogenates were prepared as described in the Methods section; protein was separated by SDS-PAGE and probed with the appropriate antibodies. GAPDH was used as an internal loading control. Intensity of the band was quantified using ImageQuant TL software and expressed as the relative ratio to loading control. (C) Caspase–1 activity was measured according to manufacturer's protocol in Sham control vs RYGB. Data are mean ± SE. * denotes a significant difference between Sham control vs. RYGB.

Western blot analysis revealed that protein expression of NLRP3 (p<0.002), ASC (p<0.00008), IL–18 (p<0.0008), caspase–1 (p<0.0003), and IL–1β (p<0.003) were significantly lower in RYGB (N = 8) than in Sham control (N = 8) in omental adipose tissue (Fig 5). Gene expression for pro-IL–1β was not significantly lower for RYGB vs. control.

### Caspase–1 activity assay

Analysis of caspase 1 activity assay of omental adipose tissue between control and RYGB group demonstrated that RYGB decreases caspase–1 activity assay (p<0.0002).

### Correlation analysis

In omental adipose tissue (Table 2), IL–6; MCP–1 and ASC gene expression were significantly correlated with both changes in body weight and changes in glucose AUC. For NLRP3, gene expression there was a significant positive correlation with changes in body weight (r = 0.48, P = 0.03) but not with glucose AUC (r = 0.35, P = 0.09). Caspase–1 was somewhat correlated with changes in body weight and significantly correlated with changes in glucose AUC. For IL–1β there was no correlation with either changes in body weight or glucose AUC. In mesenteric adipose tissue (Table 3), both IL–6 and MCP1 correlated significantly (p<0.05) with gene expression and changes in body weight, but not with changes in glucose AUC. Caspase–1 gene expression showed a significant correlation only with changes in glucose AUC. Interestingly, we did not find any significant correlation between target gene expression and improvement in body weight or glucose AUC for subcutaneous adipose tissue (Table 4).

**Table 2:** Correlation analysis between gene expression level vs. changes in body weight (left) and absolute changes of insulin resistance (right) in omental adipose tissue.

	Δ Body weight (grams)		Δ Glucose AUC (mg/dl*min)	
Gene Expression	*R*	*p Value*	*R*	*p Value*
IL–6	0.59	0.03*	0.61	0.02*
MCP1	0.54	0.01*	0.60	0.01*
IL–1β	0.01	0.48	0.25	0.18
NLRP3	0.48	0.03*	0.35	0.09
IL–18	0.46	0.03*	0.62	0.01*
ASC	0.49	0.03*	0.67	0.002*
CASP1	0.41	0.05*	0.72	0.001*

* Threshold of significance set at P<0.05

**Table 3:** Correlation analysis between gene expression, and change in body weight (left) and absolute change in insulin resistance (right) in mesenteric adipose tissue.

	Δ Body weight (grams)		Δ Glucose AUC (mg/dl*min)	
Gene Expression	*R*	*p Value*	*R*	*p Value*
IL–6	0.50	0.04*	0.26	0.17
MCP1	0.48	0.04*	0.33	0.10
IL–1β	0.40	0.09	0.05	0.43
NLRP3	0.21	0.24	0.20	0.23
IL–18	0.41	0.08	0.09	0.38
ASC	0.32	0.14	0.31	0.12
CASP1	0.33	0.13	0.60	0.01*

* Threshold of significance set at P<0.05

**Table 4:** Correlation analysis between gene expression, and changes in body weight (left) and absolute change in insulin resistance (right) in subcutaneous adipose tissue.

	Δ Body weight (grams)		Δ Glucose AUC (mg/dl*min)	
Gene Expression	*R*	*p Value*	*R*	*p Value*
IL–6	0.19	0.24	0.02	0.48
MCP1	0.05	0.43	0.03	0.46
IL–1β	0.27	0.16	0.10	0.37
NLRP3	0.02	0.47	0.19	0.27
IL–18	0.16	0.27	0.29	0.17
ASC	0.32	0.11	0.30	0.16
CASP1	0.24	0.19	0.29	0.16

* Threshold of significance set at P<0.05

## Discussion

In the present study, we assessed the effect of RYGB surgery on the expression of inflammasome components in adipose tissues of obese Sprague-Dawley rats. The major findings are:

1. Bariatric surgery reverses inflammation by suppressing NLRP3 inflammasome activation in visceral adipose tissue, and may serve as an effective anti-inflammatory treatment;
2. Bariatric surgery induces significant weight loss and improves glycemic control.

This study provides novel data on the effects of RYGB surgery on the NLRP3 inflammasome in adipose tissue (OM, MS and SQ). Using a novel approach, we examined whether the effects of bariatric surgery were associated with the regulation of inflammation in visceral (omental and mesenteric) and subcutaneous adipose tissue through the NLRP3 inflammasome. Furthermore, we studied the associations between the NLRP3 inflammasome gene expression, body weight, and glucose tolerance in obese rats. Of note, Sprague-Dawley rats are not prone to develop type 2 Diabetes, but they do become insulin resistance and experience hyperglycemia on a high fat diet.

Obesity, specifically abdominal or visceral obesity, leads to high levels of pro-inflammatory adipokines including IL–6, IL–1β, leptin and TNF-alpha. The expression and release of adipokines from adipose tissue are different in specific depot sites (omental, mesenteric, subcutaneous), with visceral fat having greater macrophage infiltration and therefore greater expression of pro-inflammatory and lower expression of anti-inflammatory adipokines than does subcutaneous fat [24], [25].

The success of bariatric surgery weight loss procedures for treating obesity and its related co-morbidities (T2D) were documented in a meta-analysis [26]. The biologic mechanisms by which bariatric surgery resolves these conditions are not fully understood. One probable mechanism is addressed by the inflammatory hypothesis, which suggests the presence of a low-grade inflammatory state in obesity [26, 27]. However, no studies to date have examined the effects of bariatric surgery on the NLRP3 inflammasome in adipose tissue from obese rats.

Previous studies demonstrated that long-term weight loss after bariatric surgery is accompanied by a decreased pro-inflammatory state, as evidenced by reduced circulating C-reactive protein (CRP) [28], [29], [30], [31], IL–6 [30] and leptin [32]. Bariatric surgery has also been shown to reduce subcutaneous adipose tissue macrophage attraction, and expression of inflammatory cytokines genes, including TNF-alpha and IL–6 [30], [33]. Also, in the setting of bariatric surgery-induced weight loss, adipose tissue macrophage infiltration decreases, glycemic status is improved [33], [34], and notably the latter correlated with decreased inflammation in our study. MCP–1 (CCL2) in adipose tissue is reduced in NLRP3 deficient mice, suggesting that NLRP3 inflammasome plays a role in macrophage infiltration in adipose tissue and correlates with sustained levels of chronic inflammation in obesity [12]. In our study, MCP–1 gene expression decreased in both visceral types of adipose tissue post-RYGB surgery—omental and mesenteric—and was positively correlated with a decrease in body weight and improvement in glycemic control. In subcutaneous fat samples, neither MCP–1 nor IL–6 gene expression correlated with any inflammasome components’ gene expression, or changes in body weight or glucose AUC. These data suggest that there is a preferential type of adipose tissue where reduction of macrophage infiltration occurs post RYGB that improves the inflammatory profile and glucose metabolism. Compared with subcutaneous fat, visceral fat (omental and mesenteric) showed a decreased transcript level for most of the NLRP3 inflammasome components, IL–6 and MCP–1 for RYGB vs. control treated groups. Also, protein levels for NLRP3, ASC, IL–18, IL1β and caspase–1 significantly decreased in omental fat in RYGB compared to the Sham control group. Similar to previously published data [35], we found that the improvements in inflammation/insulin resistance post RYGB, appear to be modulated by the inhibition of inflammation in a tissue specific manner. The roles of subcutaneous adipose tissue and visceral adipose tissue in metabolic deregulation are intrinsically different [36].

The data of Bradley et al. [37] indicate that marked weight loss in obese subjects is accompanied by changes in key parameters of postprandial glucose homeostasis, multi-organ insulin sensitivity, beta-cell function and adipose tissue inflammation. Bradley et al. also reported that weight loss following RYGB led to amelioration of pro-inflammatory cytokines, including IL–1 β. These cytokines contribute to the development of insulin resistance. IL–1 β is produced via cleavage of pro-IL–1 β by caspase–1. Our findings indicate that visceral adipose tissue expression of caspase–1 is correlated with reduction in plasma glucose in obese rats, and suggest that in adipose tissue, activation of caspase–1, possibly through NLRP3 inflammasome, during hyperglycemic conditions, leads to an enhanced production of IL–1 β that may result in the development of insulin resistance.

The relationship between the RYGB and changes in the NLRP3 inflammasome in certain depots of adipose tissue as reflected by the findings in this study, suggest that RYGB surgery decreases visceral adipose tissue inflammation and ameliorates insulin resistance, by reversing the NLRP3 inflammasome activation. An increased understanding of the pro-inflammatory stage based on NLRP3 activation may have the potential to determine optimal timing for therapeutic gastric bypass surgery. Differential expression of the NLRP3 inflammasome in adipose tissue depots suggests a new concept of tissue inflammation privilege and supports the idea that intervention at early stages of obesity, using NLRP3 inflammasome as a personalized biomarker, might prevent progression of the disease and development of co-morbidities.

## Supporting Information

S1 Table Body weight data-both groups. (PDF)

S2 Table OGTT glucose data-both groups. (PDF)

S3 Table Omental gene data. (PDF)

S4 Table Mesenteric gene data. (PDF)

S5 Table SQ gene data. (PDF)

S6 Table Caspase activity data. (PDF)

S7 Table Protein expression data—all meas. (PDF)

## References

[1] Donath MY, Shoelson SE. Type 2 diabetes as an inflammatory disease. Nat Rev Immunol. 2011;11(2):98–107. 10.1038/nri2925 21233852

[2] Kanneganti TD, Ozören N, Body-Malapel M, Amer A, Park JH, Franchi L, et al Bacterial RNA and small antiviral compounds activate caspase–1 through cryopyrin/Nalp3. Nature. 2006;440(7081):233–6. 16407888 10.1038/nature04517

[3] Sutterwala FS, Ogura Y, Szczepanik M, Lara-Tejero M, Lichtenberger GS, Grant EP, et al Critical role for NALP3/CIAS1/Cryopyrin in innate and adaptive immunity through its regulation of caspase–1. Immunity. 2006;24(3):317–27. 16546100 10.1016/j.immuni.2006.02.004

[4] Mariathasan S, Weiss DS, Newton K, McBride J, O'Rourke K, Roose-Girma M, et al Cryopyrin activates the inflammasome in response to toxins and ATP. Nature. 2006;440(7081):228–32. 16407890 10.1038/nature04515

[5] Duewell P, Kono H, Rayner KJ, Sirois CM, Vladimer G, Bauernfeind FG, et al NLRP3 inflammasomes are required for atherogenesis and activated by cholesterol crystals. Nature. 2010;464(7293):1357–61. 10.1038/nature08938 20428172 PMC2946640

[6] Aganna E, Hawkins PN, Ozen S, Pettersson T, Bybee A, McKee SA, et al Allelic variants in genes associated with hereditary periodic fever syndromes as susceptibility factors for reactive systemic AA amyloidosis. Genes Immun. 2004;5(4):289–93. 15071491 10.1038/sj.gene.6364070

[7] Martinon F, Pétrilli V, Mayor A, Tardivel A, Tschopp J. Gout-associated uric acid crystals activate the NALP3 inflammasome. Nature. 2006;440(7081):237–41. 16407889 10.1038/nature04516

[8] Pétrilli V, Dostert C, Muruve DA, Tschopp J. The inflammasome: a danger sensing complex triggering innate immunity. Curr Opin Immunol. 2007;19(6):615–22. 17977705 10.1016/j.coi.2007.09.002

[9] Wilmanski JM, Petnicki-Ocwieja T, Kobayashi KS. NLR proteins: integral members of innate immunity and mediators of inflammatory diseases. J Leukoc Biol. 2008;83(1):13–30. 17875812 10.1189/jlb.0607402 PMC3256237

[10] Zhou R, Tardivel A, Thorens B, Choi I, Tschopp J. Thioredoxin-interacting protein links oxidative stress to inflammasome activation. Nat Immunol. 2010;11(2):136–40. 10.1038/ni.1831 20023662

[11] Stienstra R, Joosten LA, Koenen T, van Tits B, van Diepen JA, van den Berg SA, et al The inflammasome-mediated caspase–1 activation controls adipocyte differentiation and insulin sensitivity. Cell Metab. 2010;12(6):593–605. 10.1016/j.cmet.2010.11.011 21109192 PMC3683568

[12] Vandanmagsar B, Youm YH, Ravussin A, Galgani JE, Stadler K, Mynatt RL, et al The NLRP3 inflammasome instigates obesity-induced inflammation and insulin resistance. Nat Med. 2011;17(2):179–88. 10.1038/nm.2279 21217695 PMC3076025

[13] Moschen AR, Molnar C, Enrich B, Geiger S, Ebenbichler CF, Tilg H. Adipose and liver expression of interleukin (IL)-1 family members in morbid obesity and effects of weight loss. Mol Med. 2011;17(7–8):840–5. 10.2119/molmed.2010.00108 21394384 PMC3146615

[14] García de la Torre N, Rubio MA, Bordiú E, Cabrerizo L, Aparicio E, Hernández C, et al Effects of weight loss after bariatric surgery for morbid obesity on vascular endothelial growth factor-A, adipocytokines, and insulin. J Clin Endocrinol Metab. 2008;93(11):4276–81. 10.1210/jc.2007-1370 18713823

[15] Konukoglu D, Uzun H, Firtina S, Cigdem Arica P, Kocael A, Taskin M. Plasma adhesion and inflammation markers: asymmetrical dimethyl-L-arginine and secretory phospholipase A2 concentrations before and after laparoscopic gastric banding in morbidly obese patients. Obes Surg. 2007;17(5):672–8. 17658029 10.1007/s11695-007-9113-3

[16] Sturm W, Tschoner A, Engl J, Kaser S, Laimer M, Ciardi C, et al Effect of bariatric surgery on both functional and structural measures of premature atherosclerosis. Eur Heart J. 2009;30(16):2038–43. 10.1093/eurheartj/ehp211 19502233

[17] Vázquez LA, Pazos F, Berrazueta JR, Fernández-Escalante C, García-Unzueta MT, Freijanes J, et al Effects of changes in body weight and insulin resistance on inflammation and endothelial function in morbid obesity after bariatric surgery. J Clin Endocrinol Metab. 2005;90(1):316–22. 15507518 10.1210/jc.2003-032059

[18] Rubino F. Is type 2 diabetes an operable intestinal disease? A provocative yet reasonable hypothesis. Diabetes Care. 2008;31 Suppl 2:S290–6. 10.2337/dc08-s271 18227499

[19] Couzin J. Medicine. Bypassing medicine to treat diabetes. Science. 2008;320(5875):438–40. 10.1126/science.320.5875.438 18436751

[20] Dixon JB, O'Brien PE, Playfair J, Chapman L, Schachter LM, Skinner S, et al Adjustable gastric banding and conventional therapy for type 2 diabetes: a randomized controlled trial. JAMA. 2008;299(3):316–23. 10.1001/jama.299.3.316 18212316

[21] Gatmaitan P, Huang H, Talarico J, Moustarah F, Kashyap S, Kirwan JP, et al Pancreatic islet isolation after gastric bypass in a rat model: technique and initial results for a promising research tool. Surg Obes Relat Dis. 2010;6(5):532–7. 10.1016/j.soard.2010.05.018 20678966

[22] Meguid MM, Ramos EJ, Suzuki S, Xu Y, George ZM, Das UN, et al A surgical rat model of human Roux-en-Y gastric bypass. J Gastrointest Surg. 2004;8(5):621–30. 15240001 10.1016/j.gassur.2004.02.003

[23] Livak KJ, Schmittgen TD. Analysis of relative gene expression data using real-time quantitative PCR and the 2(-Delta Delta C(T)) Method. Methods. 2001;25(4):402–8. 11846609 10.1006/meth.2001.1262

[24] Zoico E, Di Francesco V, Mazzali G, Vettor R, Fantin F, Bissoli L, et al Adipocytokines, fat distribution, and insulin resistance in elderly men and women. J Gerontol A Biol Sci Med Sci. 2004;59(9):M935–9. 15472159 10.1093/gerona/59.9.m935

[25] Alvehus M, Burén J, Sjöström M, Goedecke J, Olsson T. The human visceral fat depot has a unique inflammatory profile. Obesity (Silver Spring). 2010;18(5):879–83. 20186138 10.1038/oby.2010.22

[26] Buchwald H, Avidor Y, Braunwald E, Jensen MD, Pories W, Fahrbach K, et al Bariatric surgery: a systematic review and meta-analysis. JAMA. 2004;292(14):1724–37. 15479938 10.1001/jama.292.14.1724

[27] Esposito K, Giugliano G, Scuderi N, Giugliano D. Role of adipokines in the obesity-inflammation relationship: the effect of fat removal. Plast Reconstr Surg. 2006;118(4):1048–57; discussion 58–9. 16980868 10.1097/01.prs.0000232281.49432.ce

[28] Habib P, Scrocco JD, Terek M, Vanek V, Mikolich JR. Effects of bariatric surgery on inflammatory, functional and structural markers of coronary atherosclerosis. Am J Cardiol. 2009;104(9):1251–5. 10.1016/j.amjcard.2009.06.042 19840571

[29] Sledzinski T, Sledzinski M, Smolenski RT, Swierczynski J. Increased serum nitric oxide concentration after bariatric surgery–-a potential mechanism for cardiovascular benefit. Obes Surg. 2010;20(2):204–10. 10.1007/s11695-009-0041-2 19997784

[30] Moschen AR, Molnar C, Geiger S, Graziadei I, Ebenbichler CF, Weiss H, et al Anti-inflammatory effects of excessive weight loss: potent suppression of adipose interleukin 6 and tumour necrosis factor alpha expression. Gut. 2010;59(9):1259–64. 10.1136/gut.2010.214577 20660075

[31] Laimer M, Ebenbichler CF, Kaser S, Sandhofer A, Weiss H, Nehoda H, et al Markers of chronic inflammation and obesity: a prospective study on the reversibility of this association in middle-aged women undergoing weight loss by surgical intervention. Int J Obes Relat Metab Disord. 2002;26(5):659–62. 12032750 10.1038/sj.ijo.0801970

[32] Ashrafian H, le Roux CW, Darzi A, Athanasiou T. Effects of bariatric surgery on cardiovascular function. Circulation. 2008;118(20):2091–102. 10.1161/CIRCULATIONAHA.107.721027 19001033

[33] Cancello R, Henegar C, Viguerie N, Taleb S, Poitou C, Rouault C, et al Reduction of macrophage infiltration and chemoattractant gene expression changes in white adipose tissue of morbidly obese subjects after surgery-induced weight loss. Diabetes. 2005;54(8):2277–86. 16046292 10.2337/diabetes.54.8.2277

[34] Kováčiková M, Sengenes C, Kováčová Z, Šiklová-Vítková M, Klimčáková E, Polák J, et al Dietary intervention-induced weight loss decreases macrophage content in adipose tissue of obese women. Int J Obes (Lond). 2011;35(1):91–8. 20531347 10.1038/ijo.2010.112

[35] Yin D, Gao Q, Ma LL, Yan W, Williams P, Wasserman D, et al Bariatric surgery regulates tissue specific inflammation in high fat diet-induced obese mice. The Journal of Immunology. 2011;186, 50.14.

[36] Poulain-Godefroy O, Le Bacquer O, Plancq P, Lecoeur C, Pattou F, Frühbeck G, et al Inflammatory role of Toll-like receptors in human and murine adipose tissue. Mediators Inflamm. 2010;2010:823486 10.1155/2010/823486 20339530 PMC2843862

[37] Bradley D, Conte C, Mittendorfer B, Eagon JC, Varela JE, Fabbrini E, et al Gastric bypass and banding equally improve insulin sensitivity and β cell function. J Clin Invest. 2012;122(12):4667–74. 10.1172/JCI64895 23187122 PMC3512168

