# Supplementary material for: Effect of Roux-en-Y Gastric Bypass on the NLRP3 Inflammasome in Adipose Tissue from Obese Rats
Source: PLoS One. 2015 Oct 5;10(10):e0139764. doi: 10.1371/journal.pone.0139764 (PMC4593548; doi:10.1371/journal.pone.0139764)
Supplement: S1 Table — (PDF) [file pone.0139764.s001.pdf]

Body weight (grams)

Day	RYGB	Control
Baseline	336.6	326.8
0	757.2375	735.5625
7	673.3375	698.3875
14	657.45	706.175
21	652.8125	730.3875
28	643.3	747.8125
35	636.6875	761.1125
42	620.725	760.65
49	622.8875	766.075
56	614.8125	779.85
63	619	792.1625
70	622.1125	814.05
77	625.1125	839.375
84	617.075	856.2625
91	598.2625	871.7125
