# Supplementary material for: Effect of Roux-en-Y Gastric Bypass on the NLRP3 Inflammasome in Adipose Tissue from Obese Rats
Source: PLoS One. 2015 Oct 5;10(10):e0139764. doi: 10.1371/journal.pone.0139764 (PMC4593548; doi:10.1371/journal.pone.0139764)
Supplement: S2 Table — (PDF) [file pone.0139764.s002.pdf]

OGTT - AUC - SHAM

Pre-op	30 day
9560	9025
13950	10795
12875	10555
9470	9945
11940	30845
6930	8845
9985	10855
7045	10535

OGTT - AUC - RYGB

Pre-op	30 day
10955	8035
15695	14410
14985	5880
11280	9630
18030	11620
10430	9640
14770	10750
7585	6520
