# Supplementary material for: Effect of Roux-en-Y Gastric Bypass on the NLRP3 Inflammasome in Adipose Tissue from Obese Rats
Source: PLoS One. 2015 Oct 5;10(10):e0139764. doi: 10.1371/journal.pone.0139764 (PMC4593548; doi:10.1371/journal.pone.0139764)
Supplement: S3 Table — (PDF) [file pone.0139764.s003.pdf]

		Group	Fold induction (relative to co
			Group average
OM Omental AT	IL6	sham	2.645
OM Omental AT	IL6	RYGB	0.280

		Group	Fold induction (relative to co
			Group average
OM Omental AT	MCP1	sham	2.274
OM Omental AT	MCP1	RYGB	0.522

		Group	Fold induction (relative to co
			Group average
OM Omental AT	IL1 Beta	sham	1.624
OM Omental AT	IL1 Beta	RYGB	1.244

		Group	Fold induction (relative to co
			Group average
OM Omental AT	NLRP3	sham	1.874
OM Omental AT	NLRP3	RYGB	0.856

		Group	Fold induction (relative to co
			Group average
OM Omental AT	IL18	sham	2.434
OM Omental AT	IL18	RYGB	0.724

		Group	Fold induction (relative to co
			Group average
OM Omental AT	CASP1	sham	2.470
OM Omental AT	CASP1	RYGB	0.690

		Group	Fold induction (relative to co
			Group average
OM Omental AT	ASC	sham	2.316
OM Omental AT	ASC	RYGB	0.514

ntrol group)
Group sem
1.235
0.083

ntrol group)
Group sem
0.929
0.208

ntrol group)
Group sem
0.593
0.669

ntrol group)
Group sem
0.613
0.250

ntrol group)
Group sem
0.952
0.378

ntrol group)
Group sem
1.195
0.385

ntrol group)
Group sem
1.001
0.264
