# Supplementary material for: Effect of Roux-en-Y Gastric Bypass on the NLRP3 Inflammasome in Adipose Tissue from Obese Rats
Source: PLoS One. 2015 Oct 5;10(10):e0139764. doi: 10.1371/journal.pone.0139764 (PMC4593548; doi:10.1371/journal.pone.0139764)
Supplement: S4 Table — (PDF) [file pone.0139764.s004.pdf]

		Group	Fold induction (relative to
			Group average
MS Mesenteric AT	IL6	sham	2.351
MS Mesenteric AT	IL6	RYGB	0.287

		Group	Fold induction (relative to
			Group average
MS Mesenteric AT	MCP1		3.589
MS Mesenteric AT	MCP1	RYGB	0.198

		Group	Fold induction (relative to
			Group average
MS Mesenteric AT	IL1 Beta	sham	2.502
MS Mesenteric AT	IL1 Beta	RYGB	0.479

		Group	Fold induction (relative to
			Group average
MS Mesenteric AT	NLRP3	sham	1.954
MS Mesenteric AT	NLRP3	RYGB	0.998

		Group	Fold induction (relative to
			Group average
MS Mesenteric AT	IL18	sham	2.938
MS Mesenteric AT	IL18	RYGB	0.258

		Group	Fold induction (relative to
			Group average
MS Mesenteric AT	CASP1	sham	3.442
MS Mesenteric AT	CASP1	RYGB	0.228

		Group	Fold induction (relative to
			Group average
MS Mesenteric AT	ASC	sham	4.068
MS Mesenteric AT	ASC	RYGB	0.410

control group)
Group sem
1.130
0.206

control group)
Group sem
1.592
0.073

control group)
Group sem
1.184
0.154

control group)
Group sem
0.981
0.173

control group)
Group sem
1.451
0.072

control group)
Group sem
2.143
0.093

control group)
Group sem
2.514
0.106
