# Supplementary material for: Effect of Roux-en-Y Gastric Bypass on the NLRP3 Inflammasome in Adipose Tissue from Obese Rats
Source: PLoS One. 2015 Oct 5;10(10):e0139764. doi: 10.1371/journal.pone.0139764 (PMC4593548; doi:10.1371/journal.pone.0139764)
Supplement: S5 Table — (PDF) [file pone.0139764.s005.pdf]

		Group	Fold induction (relative to control group)	
			Group average	Group sem
SQ Subcutaneous AT	IL6	sham	3.363	1.136
SQ Subcutaneous AT	IL6	RYGB	11.746	5.945

		Group	Fold induction (relative to control group)	
			Group average	Group sem
SQ Subcutaneous AT	MCP1	sham	1.832	0.594
SQ Subcutaneous AT	MCP1	RYGB	2.509	1.032

		Group	Fold induction (relative to control group)	
			Group average	Group sem
SQ Subcutaneous AT	IL1 Beta	sham	0.610	0.310
SQ Subcutaneous AT	IL1 Beta	RYGB	4.974	3.290

		Group	Fold induction (relative to control group)	
			Group average	Group sem
SQ Subcutaneous AT	NLRP3	sham	1.300	0.340
SQ Subcutaneous AT	NLRP3	RYGB	1.484	0.294

		Group	Fold induction (relative to control group)	
			Group average	Group sem
SQ Subcutaneous AT	IL18	sham	2.040	0.989
SQ Subcutaneous AT	IL18	RYGB	1.923	0.843

		Group	Fold induction (relative to control group)	
			Group average	Group sem
SQ Subcutaneous AT	CASP1	sham	3.357	1.647
SQ Subcutaneous AT	CASP1	RYGB	2.228	0.650

		Group	Fold induction (relative to control group)	
			Group average	Group sem
SQ Subcutaneous AT	ASC	sham	2.735	1.114
SQ Subcutaneous AT	ASC	RYGB	1.708	0.555
