# Supplementary material for: Effect of Roux-en-Y Gastric Bypass on the NLRP3 Inflammasome in Adipose Tissue from Obese Rats
Source: PLoS One. 2015 Oct 5;10(10):e0139764. doi: 10.1371/journal.pone.0139764 (PMC4593548; doi:10.1371/journal.pone.0139764)
Supplement: S6 Table — (PDF) [file pone.0139764.s006.pdf]

Caspase 1 activity assay (units/mg protein)

	average	sem
OM - Sham	1.926	0.118
OM - RYGB	1.739	0.038
