# Supplementary material for: Effect of Roux-en-Y Gastric Bypass on the NLRP3 Inflammasome in Adipose Tissue from Obese Rats
Source: PLoS One. 2015 Oct 5;10(10):e0139764. doi: 10.1371/journal.pone.0139764 (PMC4593548; doi:10.1371/journal.pone.0139764)
Supplement: S7 Table — (PDF) [file pone.0139764.s007.pdf]

Caspase-1

	average	error
sham	0.265	0.055
RYGB	0.178	0.053

NLRP3

	average	error
sham	1.114	0.189
RYGB	0.486	0.129

ASC

	average	error
sham	1.157	0.298
RYGB	0.226	0.089

IL-18

	average	error
sham	1.125	0.352
RYGB	0.202	0.091

pro IL-1β

	average	error
Sham	1.000	0.455
RYGB	0.878	0.426

IL-1β

	average	stdev
Sham	1.000	0.354
RYGB	0.768	0.464
